# A future directions of renal cell carcinoma treatment: combination of immune checkpoint inhibition and carbon ion radiotherapy

**DOI:** 10.3389/fimmu.2024.1428584

**Published:** 2024-07-18

**Authors:** Zhouhang Zheng, Tianci Yang, Yixuan Li, Pei Qu, Zhiang Shao, Yuan Wang, Wei Chang, Shahzad Muhammad Umar, Jufang Wang, Nan Ding, Wei Wang

**Affiliations:** ^1^ Department of Urology, The Second Hospital & Clinical Medical School, Lanzhou University, Lanzhou, Gansu, China; ^2^ Key Laboratory of Space Radiobiology of Gansu Province & Key Laboratory of Heavy Ion Radiation Biology and Medicine of Chinese Academy of Sciences, Institute of Modern Physics, Chinese Academy of Sciences, Lanzhou, China; ^3^ College of Life Science, University of Chinese Academy of Sciences, Beijing, China

**Keywords:** renal cell carcinoma (RCC), radiotherapy, immunotherapy, carbon ion radiotherapy, combination therapy

## Abstract

Renal cell carcinoma (RCC) is considered radio- and chemo-resistant. Immune checkpoint inhibitors (ICIs) have demonstrated significant clinical efficacy in advanced RCC. However, the overall response rate of RCC to monotherapy remains limited. Given its immunomodulatory effects, a combination of radiotherapy (RT) with immunotherapy is increasingly used for cancer treatment. Heavy ion radiotherapy, specifically the carbon ion radiotherapy (CIRT), represents an innovative approach to cancer treatment, offering superior physical and biological effectiveness compared to conventional photon radiotherapy and exhibiting obvious advantages in cancer treatment. The combination of CIRT and immunotherapy showed robust effectiveness in preclinical studies of various tumors, thus holds promise for overcoming radiation resistance of RCC and enhancing therapeutic outcomes. Here, we provide a comprehensive review on the biophysical effects of CIRT, the efficacy of combination treatment and the underlying mechanisms involved in, as well as its therapeutic potential specifically within RCC.

## 1 Introduction

Globally, renal tumors are the 14^th^ most prevalent malignant tumors, registering 430,000 new cases in 2020. With a steadily rising incidence rate, the burden of RCC continues to grow, resulting in numerous patients dying annually due to inadequate treatment (1, 2). 90% of renal tumors are renal cell carcinoma (RCC). At the time of diagnosis, 30% of RCC patients already exhibit metastasis, and a prognostic analysis indicates that nearly 30% of postoperative patient possess recurrence or metastasis. Since RCC responds poorly to both radiation and chemotherapy, a more effective treatment is critical for current RCC therapy (3–5).

Immune checkpoint inhibitors (ICIs), such as anti-programmed death receptor 1 (anti-PD-1), anti-programmed death-ligand 1 (anti-PD-L1), and anti-cytotoxic T-lymphocyte-associated protein-4 (anti-CTLA-4), constitute the primary approach to immunotherapy, achieving a considerable effect in patient with non-small cell lung cancer (NSCLC) or malignant melanoma among other cancer patient (6, 7). For RCC, ICIs treatment notably enhances the objective response and overall survival rates in patients with advanced disease, serving as the first- and second-line therapies for metastatic RCC. However, the total clinical efficacy rate stands at roughly 20%-40%, with only a subset of patients truly benefiting from immunotherapy (8–10). Hence, augmenting the effects of immunotherapy especially a combining treatment of immunotherapy with different therapeutic strategies is the major concern of RCC therapy currently. Studies indicate that RT can modulate immune responses and induce immunogenic cell death, thereby enhancing the systemic anti-tumor effects of immunotherapy. Extensive research focusing on the combination of RT with ICIs is underway (11, 12). Since RCC exhibits limited sensitivity to X-ray and γ-ray radiation, few studies reported the application of combination treatment in RCC.

Charged particles, especially hydrogen ions (protons) and carbon ions, are being increasingly used for cancer therapy. Notably, carbon ion radiotherapy (CIRT), the most prevalent used heavy ion radiation, has superior physical and biological characteristics to conventional photon radiation such as X-rays or γ-rays (13, 14). In addition, the combination treatment of CIRT and immunotherapy of malignant melanoma and breast cancer showed a better tumor suppression effect than the combination treatment of proton radiation thus present an encouraging prospect of CIRT combination treatment (13, 15). The combination treatment of CIRT and ICIs might offer a solution to RCC therapy.

In this article, we reviewed the biophysical effects of CIRT, the efficacy of combination treatment and the underlying mechanisms involved in, as well as its therapeutic potential specifically within RCC.

## 2 Radiation therapy and radiation therapy of RCC

### 2.1 Radiation therapy and CIRT

Approximately half of global tumor patients are treated with ionizing radiation (IR) therapy nowadays, either alone or as a complement to other treatments (16). Photon radiation such as X-rays and γ-rays are the most common cancer treatments and recently RT has emerged as a primary approach for cancer treatments (17). Though clinical photon radiotherapy enables the effective dosage distribution of tumor area, the concurrently irradiation of surrounding healthy tissues is inevitable and nonnegligible. Clinically, the late effects of radiotherapy gravely impact patients’ quality of life. Consequently, an important aspect of current radiation oncology research is still focusing on strategies to mitigate the influence of conventional radiation therapy on healthy tissues by other treatments such as chemotherapy, surgery, immunotherapy, or comprehensive treatments.

Preclinical studies and clinical usage of particle radiation, such as protons, heavy ions beam, neutrons, and boron neutrons therapy, indicate its distinct physical and biological attributes. Presently, carbon ion beams represent the most prevalent types of medical heavy ions beam radiation and its elevated linear energy transfer (LET) endows it with distinctive radiobiological traits. When carbon ions enter tissue, they will release the vast majority of their energy at the end of trajectory, form an inverted energy deposition curve which called Bragg peak (18). Modulating the energy of ions allows for adjusting the depth of the Bragg peak in tissue, leading to the focused release of energy within tumor sites. This characteristic of energetic charge particle radiation greatly reduces energy deposition in healthy tissues, providing precise targeting of tumor area and minimizing side effects. Yet, the Bragg peak’s duration is sharp, with its half-maximal width spanning just a few millimeters. In clinical settings, a spread-out Bragg peak (SOBP) is frequently utilized to encompass tumor areas. The high linear energy transfer (LET) characteristic ensures that the SOBP has elevated radiation dose deposition, facilitated accurate and thorough lesion irradiation while also significantly reduced harm to healthy tissue (19). Additionally, CIRT typically causes clustered DNA damage, making it difficult for cells to repair, thus facilitating the elimination of tumor cells (20). Furthermore, CIRT is less sensitive to the alterations in tumor cell cycle, radiation susceptibility, and oxygen concentration, allowing them to substantially counteract tumor radio-resistance.

Currently, the US, Japan, Germany, and China are leading in the field of heavy ion cancer therapy, achieving significant results in breast cancers, skin cancer, cranial cancers, prostate cancer, and more. In China, several hospitals now offer heavy ion cancer treatment services. For instance, the The Wuwei Tumor Hospital in northwest China’s Gansu Province boasts China’s first self-developed carbon ion therapy facility which was co-developed by the Institute of Modern Physics, Chinese Academy of Sciences (CAS) and a subsidiary company. Since 2020, the facility has completed over 1,500 cases of tumor patient, achieving considerable success. According to the data from the Particle Therapy Co-Operative Group (PTCOG), by the end of 2020, 40,000 patients globally had undergone heavy ion radiotherapy (primarily CIRT).

Currently, many clinical studies on heavy ion radiotherapy have been completed (**Table 1**) or are in progress (**Table 2**). Among 216 NSCLC patients, single-fraction CIRT showed comparable efficacy to the previous fractionated regimens and only one case of serious late toxicity was observed (21). 21 patients with melanoma treated with heavy ion beam irradiation showed that the 3-year local control rate was 92.3% (22). 75 patients with locally recurrent nasopharyngeal carcinoma were treated with CIRT, the 1-year overall survival was 98.1% and no severe acute or late toxicities were found (23). Among 2,157 prostate cancer patients receiving CIRT, the 5-year biochemical relapse-free survival rates for low, intermediate, and high-risk patients were 100%and no serious toxicities were identified (24). In addition, in cervical cancer, uterine adenocarcinoma, rectal cancer, hepatocellular carcinoma, chondrosarcoma, pancreatic cancer, and chordoma, heavy ion radiotherapy has also shown better clinical results and lower radiation toxicities (25–29). As compared with XRT, the 5-year overall survival rate for malignant melanoma post-CIRT (44%) was significantly higher than conventional radiation therapy (CRT) (25%) (30); In stage I inoperable NSCLC patients, the 5-year overall survival rate for CIRT (42%) was significantly higher than CRT (20%) (31). All these studies demonstrated the safety and efficacy of heavy ion radiotherapy especially the CIRT.

**Table 1:** Clinical cases of heavy-ion radiotherapy.

Tumor types	Sample size	Dosage	Efficacy	Toxicity
nasopharyngeal carcinoma^1^	75	50-66 Gy, 2-3 Gy/fraction	1-year OS: 98.1%	No≥grade 2 acute toxicity; ≥grade 3 late toxicity: mucosal necrosis (9.3%) dry mouth (1.3%) temporal lobe necrosis (1.3%)
Melanoma^2^	21	57.6-64 Gy/16 fractions	3-year OS:49.2% 3-year LCR:92.3%	Acute grade 2-3 toxicity: mucositis (53%), leukopenia (43%) was improved after conservative treatment no ≥grade 3 late toxicities
prostate cancer^3^	2517	63 Gy、66 Gy/20 fractions 57.6 Gy/16 fractions 51.6 Gy/12 fractions	5-year OS: Low risk group,100%; medium risk group,99%; high risk group,96% 5-year LCR: low risk group,98%; medium risk group: 96%; high risk group: 99%	Grade 2 late toxicity: GU 4.6%、GI 0.4% No ≥ grade 3 GI toxicity ≥ grade 3 acute and late GU Toxicity (<0.1%) grade 2 late toxicity: GU 4.6%, GI0.4%
NSCLC^4^	216	28 Gy-50 Gy	5-year OS:49.4%; 5-year LCR:72.7%	No ≥grade 3 toxicities (pulmonary and skin adverse effects); grade 2 toxicities (< 2%) 1 case of grade 3 late toxicity (chest wall pain)
adenocarcinoma of the uterine cervix^5^	58	Total pelvic irradiation:36 Gy/12 fractions, Local enhanced irradiation:26.4 Gy-38.4 Gy/8 fractions	5-year OS (Salvage Surgery):68.2% 5-year OS:38.1% 5-year LCR:54.5%	≥Grade 2 late toxicity (13.8%)
Rectal Cancer^6^	180	67.2-73.6 Gy/16 fractions	73.6Gy: 5-year OS:59%; 5-year LCR:88%. Single-arm phase 2 trial: 3-year OS: 75%;	No > grade 3 acute toxicity
Uterine Cervical Carcinoma^7^	22	72 Gy/20 fractions	2-year OS:82%; 2-year LCR:67%. 2-year LCR (Salvage therapy): 81%	Acute toxicity: stage 1, no ≥grade 3 non- hematologic toxicity; stage 2, grade 3 diarrhea (1 case), grade 3 nausea (2 cases), no acute grade 4 hematologic toxicity late toxicity: ≤grade 3 gastrointestinal toxicity (2 cases)
hepatocellular carcinoma^8^	23	55、60、65 and 70 Gy/10 fractions	1-year OS:91.3% 3-year OS:81.9% 5-year OS:67.1%	Acute toxicity: grade 3 toxicity 8.7% (leukopenia) late toxicity: grade 4 8.7% (due to gastric bleeding, cirrhosis and portal hypertension)
chordomas and chondrosarcomas^9^	67	60 Gy/20 fractions	Chondrosarcoma: 3-year OS:100%, 3-year LC: 100% Chordomas: 3-year OS:89%, 3-year LC:87%	No ≥ grade 3 toxicity
RCC^10^	10	72 Gy/16 fractions	5-year OS: 74% 5-year LCR:100%	Grade 4 skin toxicity:one patient (T4 tumor)

OS, overall survival; LCR, local control rate; GU, genitourinary toxicity; GI, Gastrointestinal toxicity; NSCLE, non-small cell lung cancer; RCC, renal cell carcinoma.

**Table 2:** Ongoing clinical trials of heavy ion radiotherapy (data from ClinicalTrials.gov).

NCT Number	Conditions	Status	Interventions	Phase:	Population	Locations
NCT05009446	•Mucosal Melanoma •Sinonasal Melanoma	Recruiting	intensity-modulated radiation therapy or volume of rotating intensity-modulated radiotherapy or Proton or heavy ion radiation therapy	Early Phase 1	28	•Eye& ENT Hospital, Fudan University, Shanghai, Shanghai, China
NCT04143984	• Nasopharyngeal Carcinoma	Recruiting	Carbon-ion radiotherapy	Phase 2	146	•Shanghai Proton and Heavy Ion Center, Shanghai, Shanghai, China
NCT02739659	•Prostate Carcinoma	Recruiting	carbon-ion radiotherapy	Not Applicable	73	• Shanghai Proton and Heavy Ion Center, Shanghai, Shanghai, China
NCT04724577	• Radiotherapy •Prostate Cancer	Recruiting	carbon ion radiotherapy	Not Applicable	30	• Shanghai Proton and Heavy Ion Center, Shanghai, China
NCT05613452	•Non-small Cell Lung Cancer	Recruiting	Carbon ion radiotherapy	Phase 2	43	• Shanghai Proton and Heavy Ion Center, Shanghai, Shanghai, China
NCT05692661	• Triple Negative Breast Cancer •HER2-positive Breast Cancer	Recruiting	proton plus carbon ion radiotherapy	Phase 1	2	• Shanghai Proton and Heavy Ion center, Shanghai, China
NCT05106699	•Prostate Cancer	Recruiting	•Radiation: proton plus carbon ion radiation	Not Applicable	54	• Shanghai Proton and Heavy Ion Center, Shanghai, China
NCT05692674	•Breast Cancer	Recruiting	adjuvant hypofractionated intensity-modulated proton radiotherapy	Phase 2	67	• Shanghai Proton and Heavy Ion center, Shanghai, China
NCT05212857	•Prostate Cancer	Recruiting	Radiotherapy for primary lesion. Radiotherapy for metastatic lesion	Phase 2	160	•Fudan University Shanghai Cancer Center, Shanghai, Shanghai, China
NCT05010343	•Localized Prostate Cancer	Recruiting	carbon ion irradation.Carbon Ion Irradiation With SIB	Phase 2	140	• Shanghai Proton and Heavy Ion Center, Shanghai, China
NCT04214366	•Adenoid Cystic Carcinoma	Recruiting	Carbon ion irradiation.Bimodal irradiation	Phase 2	314	• University of Heidelberg, Radiooncology, HIT, Heidelberg, Germany

### 2.2 Radiation therapy of RCC

*In vivo* and *in vitro* studies have confirmed that RCC is a radio-resistant tumor, and photon radiation is primarily used for symptom relief in patients with advanced renal cell carcinoma (32–34).

Recently, Stereotactic Body Radiation Therapy (SBRT) has shown intriguing therapeutic effects in the treatment of RCC (35). SBRT is a radiotherapy technique that utilizes image-guided technology and highly conformal doses, achieving significant local control rates for primary and metastatic tumors. It can be applied to patients who are not surgical candidates as well as those with extensive or oligometastatic disease. Several studies have already confirmed the excellent efficacy and safety of SBRT in RCC (36, 37), considering the side effect of photon radiation, more attempts based on different radiation strategies would still be worthful.

There are few reports of RCC heavy ion radiotherapy, but the two papers showed that CIRT has notable therapeutic effects and lower toxicity. One study exhibited intriguing outcomes. Long-term follow-up revealed that the 5-year local control rate, disease-free survival rate, tumor-specific survival rate, and overall survival rate were 94.1%, 68.9%, 100% and 89.2%, respectively (38). In another study, 10 RCC patients treated with CIRT achieved 100% local control and 74% overall survival at 5 years, with only one patient experiencing grade 4 skin toxicity. These studies indicated the safety and favorable therapeutic results of CIRT in RCC (39).

Overall, though research on CIRT for RCC is quite limited, given the biological advantages of CIRT over CRT, it holds application benefits and research value in the treatment of RCC.

## 3 Immunotherapy and ICIs in RCC

### 3.1 immunotherapy

Immunotherapy aims to enhance a patient’s own immune system’s ability to recognize and attack cancer cells, thus achieving a better tumor control effect. This approach has significantly improved the current status of cancer treatment (40). Current immunotherapeutic strategies mainly include ICIs, adoptive T cell therapy, and cancer vaccines (41). Immune checkpoints refer to a set of negative regulatory molecules owned by immune cells. Among them, the immunosuppressive transmembrane protein PD-1 is expressed on T cells, B cells and NK cells, with PD-L1 and PD-L2 being its ligands (42). Within the Tumor Microenvironment (TME), tumor cells can upregulate the expression of PD-L1 or PD-L2, by which binding to PD-1 on the surface of immune cells, thereby inducing apoptosis of immune cells and keeping surviving of themselves (43, 44). Another immune checkpoint, CTLA-4 (CD152), is a transmembrane protein expressed on CD4^+^ T cells, CD8^+^ T cells and Treg cells, with its ligands primarily being CD80 and CD86 expressed on Antigen-Presenting Cells (APCs). When CTLA-4 binds to its ligands, it promotes Treg cells to secrete immunosuppressive factors, thereby inhibiting T cell responses (45).

**Figure 1:**
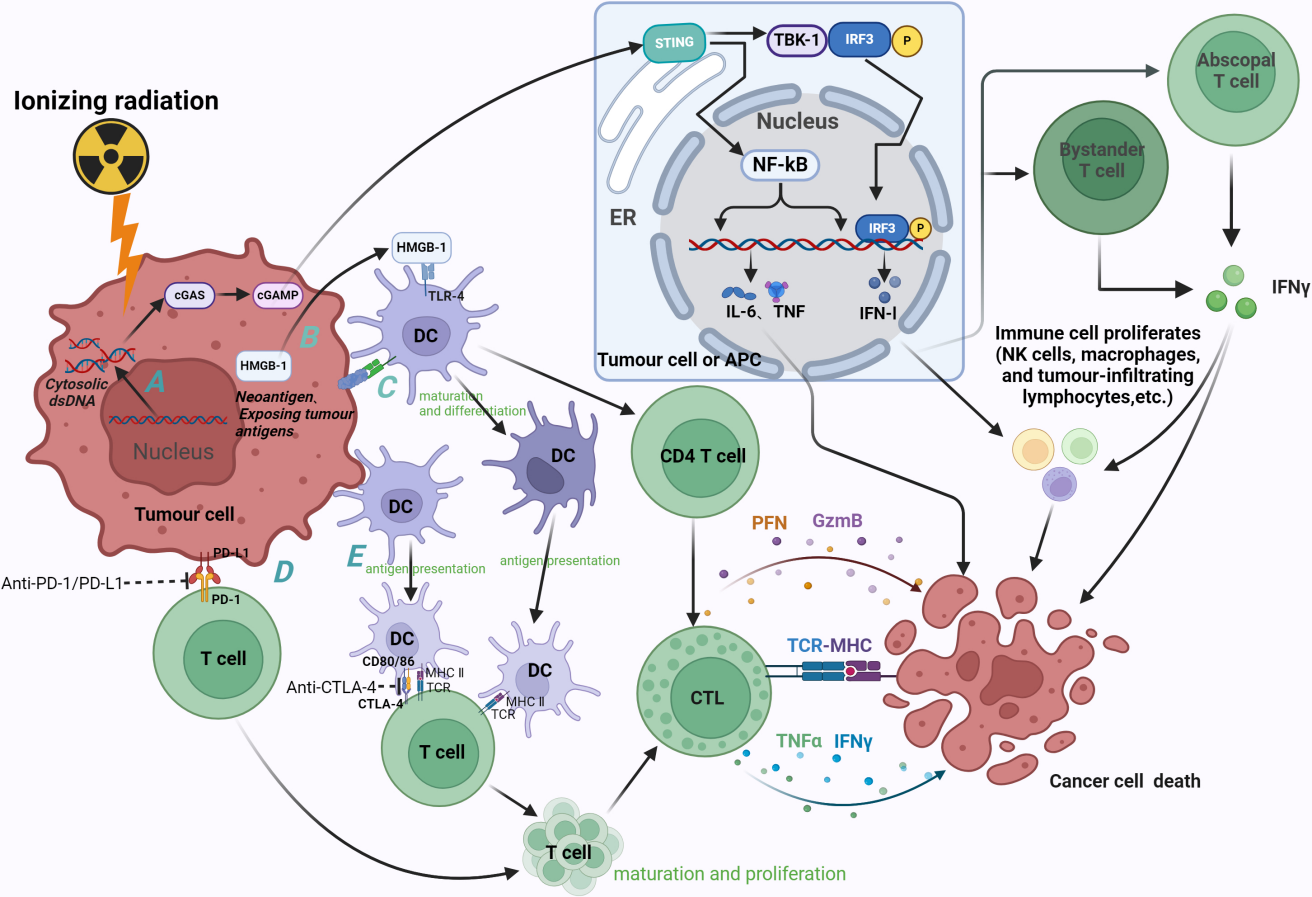
Radiation-induced immune effects and therapeutic mechanism of ICIs (anti-PD-1, anti-PD-L1 and anti-CTLA-4). **(A)** Radiation causes accumulation of cytoplasmic dsDNA in tumor cells, which can induce activation of the cGAS-STING pathway in tumor cells and APC. The enrichment of cGAS promotes the synthesis of second messenger cGAMP, which in turn binds to STING, recruits and phosphorylates downstream TBK-1 and IRF-3. Nuclear translocation of IRF-3 induces the transcription of IFN-1, and IFN-β stimulates the release of IFN-γ by bystander T cells and distant T cells. IFN can inhibit tumor cell division while inducing apoptosis, activate effector cells and increase the activity of NK cells, macrophages and tumor infiltrating lymphocytes, thereby enhancing the anti-tumor immune effect. STING induces tumor cell death by promoting the secretion of pro-inflammatory cytokines such as IL-6 and TNF through NF-kB. **(B)** Irradiation promotes HMGB-1 efflux, and HMGB-1 binds to TLR-4 to promote the differentiation and maturation of DC and antigen cross-presentation, thereby activating CD4 T cells and CTL. CTL induces apoptosis of tumor cells through secretion of PFN, GzmB, etc., or recognition of tumor cell MHC by TCR. **(C)** Radiation induces the exposure of tumor antigens and the generation of neoantigens (as indicated by calreticulin exposure to tumor cells), thereby exerting anti-tumor effects through DCs. **(D)** Irradiation can up-regulate the expression of PD-1 on immune cells and PD-L1 on tumor cells, and the combination of the two can promote the apoptosis of immune cells, resulting in immunosuppression. Anti-pd-1 and anti-PD-L1 can promote the proliferation and differentiation of immune cells (mainly T cells) by antagonizing this process, and finally play an anti-tumor role. **(E)** The immune checkpoint CTLA-4 expressed on T cells exerts immunosuppressive effects by binding to its ligands CD80 and CD86, which are mainly expressed on DC. Blockade of this process by anti-CTLA-4 can promote the proliferation and maturation of T cells. cGAS, cGMP-AMP synthase; STING, interferon gene stimulator; TBK-1, TANK-binding kinase 1; IRF-3, interferon regulatory factor 3; IFN, interferon; IL, interleukin; TNF, tumor necrosis factor; HMGB1, human high mobility group protein; TLR-4, Toll-like receptor 4; CTL, cytotoxic T cells; PFN, perforin; GzmB, Granzyme B; TCR, T cell receptor; MHC, major histocompatibility complex; ER, endoplasmic reticulum. Image created with BioRender.com, with permission.

ICIs aim to block the inhibition of immune cells to increase the number of anti-tumor cells in the TME (mainly T cells) to achieve tumor suppression (**Figure 1**). In recent years, many immunotherapeutic drugs have received FDA approval, such as anti-PD-1, anti-PD-L1, and anti-CTLA-4 monoclonal antibodies (e.g., Pembrolizumab, Atezolizumab, Nivolumab, and Ipilimumab). Clinically, ICIs have been widely used and have achieved excellent results in cancer treatment. The approval of these drugs indicates the efficacy and prospects of immunotherapy. Although immunotherapy has achieved these successes, the efficacy of monotherapy is limited, and its mechanism is not yet fully understood (46). How to make it effective for more types of tumors and benefit more patients remains an unresolved issue.

### 3.2 ICIs in RCC

Higher expression levels of PD-L1 in tumor often categorized it as an immune “hot” tumor which is inclined sensitive to ICIs (47). A majority of RCC subtypes, including clear cell RCC (ccRCC) which constitutes approximately 75% of RCC, papillary RCC, translocation RCC, and collecting duct carcinoma, exhibit a PD-L1 expression ratio exceeding 10% (48, 49). Concurrently, the preclinical use of ICIs (anti-CTLA-4 and anti-PD-1) alone or in combination in treating ccRCC has shown notable results (50–53), as illustrated in **Table 3**. Unfortunately, in clinical, RCC often develops resistance to first-line ICIs treatments, and the majority of RCC patients do not derive long-term benefits from them. Therefore, developing new treatment modalities to overcome these resistance mechanisms is crucial for the treatment of RCC.

**Table 3:** Comparison of pivotal phase III clinical trials with available results evaluating ICIs.

Author	Tested Drugs	Comparison	Phase	Histology	OS (HR, 95% CI, p)	Median PFS (HR, 95% CI, p)
Brian^1^	atezolizumab plus bevacizumab	sunitinib	III	ccRCC	24-mo: PD-L1 positive population:66%vs57%(0.84,0.62-1.15,0.2857) ITT population:63%vs60%(0.93,0.76-1.14, 0.4751)	PD-L1 positive population:11.2-mo vs 7.7-mo(0.74,0.57-0.96,0.0217) ITT population:11.2-mo vs 8.4-mo(0.83,0.70-0.94,0.0219)
Robert^2^	atezolizumab plus bevacizumab	sunitinib	III	ccRCC	PD-L1 positive population:38.7-mo(53.9%)vs 31.6-mo(56.5%),(0.85,0.64-1.13) ITT population:36.1-mo(54.8%)vs35.3-mo(55.3%), (0.91,0.76-1.08,0.27)	PD-L1 positive population: 8.9-mo vs 7.2-mo(0.93,0.72-1.21, 0.6138) ITT population:9.6-mo vs 8.3-mo(0.88,0.74-1.04,0.1218)
Brian^3^	atezolizumab plus axitinib	sunitinib	III	ccRCC	12-mo: 82.3%vs72.1%(0.53, 0.38-0.74, P<0.0001)	15.1-mo vs 11.1-mo(0.69, 0.57- 0.84, P<0.001
Robert^4^	nivolumab plus cabozantinib	sunitinib	III	ccRCC	24-mo:70%vs60%(0.70,0.55-0.9,0.0043)	16.8-mo vs 8.3-mo(0.56,0.46-0.68, p<0.0001)

OS, overall survival; PFS, progression-free survival; HR, Hazard Ratio; ccRCC, clear cell renal cell carcinoma; mo, months.

Considering the limitations of monotherapy with immunotherapy, researchers have become increasingly interested in combination strategies of ICIs with other treatment modalities. Among them, RT has garnered significant attention due to its immune-stimulating effects, and numerous related studies have been conducted. However, there is limited research on the combination treatment of ICIs and RT in RCC. The radio-resistance of RCC might be one of the factors limiting work in this area. CIRT demonstrates superior efficacy in RCC compared to CRT. The future utilization of CIRT is anticipated to be extensive in the management of RCC. Considering the commendable efficacy demonstrated by ICIs in RCC treatment, the amalgamation of CIRT and ICIs has the potential to further augment the anti-tumor effect exerted by ICIs. This combination warrants further investigation in subsequent studies. CIRT has gained attention for its immune-stimulating effects in RCC. The synergy between CIRT and ICIs holds promise for enhancing anti-tumor effects, emphasizing the need for further investigation in future studies.

## 4 The combination of RT and immunotherapy

Radiation causes direct damage to tumor cells while also inducing systemic biological effects that generate anti-tumor responses (**Figure 1**). Through these mechanisms, RT can transform tumors with low immune cell infiltration (referred to as “cold” tumors) into tumors with high immune cell infiltration (referred to as “hot” tumors), thereby enhancing the effect of tumor immunotherapy (54). This role of RT has been widely recognized, forming the fundamental principle behind the combination of radiation and immunotherapy.

### 4.1 The combination of CRT and immunotherapy

Current RT combined with immunotherapy includes combinations with DC vaccine, ICIs, and CAR-T cell therapy. Nesslinger et al. assessed the serum samples of prostate cancer patients who received cancer vaccine combined with RT or RT alone. The results indicated that the combination therapy enhanced the spread of antigens and the immune response to other tumor antigens (55). In a Phase II clinical trial, most prostate cancer patients who received cancer vaccine combined with RT showed a significant increase in PAS-specific T cells, indicating a PAS-specific cellular immune response, which was not observed in the solo radiotherapy group (56). A meta-analysis revealed that the combination of PD-1/PD-L1 inhibitors with RT extended the overall survival of NSCLC patients, simultaneously improving the objective response rate and disease control rate, without increasing the incidence of adverse events above grade 3. It was also found that SBRT or SBRT combined with surgery and PD-1/PD-L1 inhibitors is more effective than CRT combined with PD-1/PD-L1 inhibitors (57). Formenti et al. confirmed that in chemo-resistant metastatic NSCLC, RT combined with CTLA-4 inhibitor induced systemic anti-tumor T cells. Treatment with anti-CTLA-4 antibodies alone or in combination with chemotherapy was less effective (58). DeSelm et al. found that low-dose radiation sensitized tumor cells to the immune rejection response of locally activated CAR-T cells, reducing the recurrence of antigen-negative tumors (59). Current research in the CAR-T cell and radiation fields is limited, but it’s clear that RT can play an essential role in the combination treatment of patients undergoing CAR-T therapy in various clinical settings (60). However, RT also has a negative immune regulatory effect, promoting immune suppression to some extent. When radiation kills tumor cells, it can also cause a certain degree of lethal radiation. The local hypoxic environment caused by RT induces the aggregation of immune suppressive cells and M2-type macrophages in the TME and the elevation of immune suppressive factors.

In summary, RT, as a local treatment modality, can trigger a systemic immune response, suppressing the growth of distant non-irradiated tumors, also known as the abscopal effect. This became the fundamental principle of combining RT and immunotherapy. However, it can also lead to immune suppression. Therefore, when RT triggers a systemic anti-tumor immune response, administering immunotherapy as an adjunct to counteract the negative immune regulatory effects of RT and amplify its immune-stimulating effects holds promise as a curative treatment for tumors and achieving control over systemic metastatic tumors.

### 4.2 The combination of CIRT and immunotherapy

Carbon ion has unique biological and physical efficacy compared to CRT (61). Compared to X-rays, carbon ions have a stronger cytotoxic effect on tumor cells, and the enhancement of radiotherapy-induced immune cell infiltration and remodeling of the TME is more pronounced. For deep tumors like RCC that are resistant to CRT, carbon ions achieve a cytotoxic effect that CRT cannot achieve and can effectively promote immune responses. Thus, the application of “CIRT combining immunotherapy” shows a very promising prospect. Currently, the combination of CIRT and immunotherapy has made some progress in NSCLC (62), and this combined approach is increasingly being seen as an effective cancer treatment modality.

Carbon ions induce immune stimulation. Research has found that carbon ion beams can regulate anti-tumor immunity in various ways and have unique advantages in remodeling the TME. A large amount of literature has reported the advantages of carbon ion beams in immune regulation. Spina et al. compared the immune regulatory effects of CIRT and biologically equivalent doses of X-ray radiotherapy (XRT) in an *in situ* 4T1 breast cancer mouse model. The results found that low-dose CIRT can usually retain lymphocytes that are crucial for the anti-tumor immune response, and higher dose CIRT more effectively induced the secretion of pro-inflammatory cytokines, while even at low doses, XRT had lymphotoxicity (15). Guo et al.’s study showed that the activation of the cGAS-STING pathway caused by CIRT is greater than that of XRT, and it also promoted the infiltration of NK cells, CD4^+^ and CD8^+^ T cells (63). In Luo et al.’s research, CIRT was able to reduce the phosphorylation of STAT3 mediated by the JAK3/STAT2 pathway, thereby inhibiting the formation of regulatory T cells (Treg) (64), and in TME, Tregs can suppress the anti-tumor activity of NK cells (65). In a study of 32 localized prostate patients treated with CIRT, it was found that the carbon ion could effectively retain lymphocyte numbers, promote their proliferation, enhance T-cell function, and also weaken the induction of immunosuppressive cells and the expression of immunosuppressive cell factors, thus effectively inducing immune activation (66). These results indicate that compared to XRT, CIRT can significantly promote the proliferation of immune cells and the secretion of pro-inflammatory cytokines in both animals and humans, exerting a more effective anti-tumor effect.

Carbon ion beams have a superior ability to induce immunogenic cell death compared to CRT. While irradiating and killing tumor cells, they also induce immunogenic changes in these cells, which is one of the key mechanisms by which radiotherapy modulates anti-tumor immunity. Ran et al. discovered that after XRT and CIRT treatments, A549, H520, and Lewis Lung Carcinoma (LLC) cells both showed time-dependent increases in the levels of HMGB1, IL-10, and TGF-β. However, only XRT exhibited a dose-dependent increase in HMGB1 levels. Simultaneously, carbon ions induced a higher level of HMGB1, while relatively reducing the levels of immunosuppressive factors IL-10 and TGF-β (67). Onishi M and colleagues irradiated HeLa, SiHa and KYSE-70 cells with carbon ions of different LET, finding that HMGB1 release levels significantly increased in all these cancer cell lines and intensified with increasing LET (68). HMGB1, upon binding with TLR-4, promotes cytokine secretion, antigen cross-presentation, and the differentiation and maturation of DC, further activating helper and effector T cells (69); TGF-β and IL-10 are significant immunosuppressive factors in the TME, working by reducing DC function and T-cell activation, promoting Treg cell transformation, and facilitating MDSC maturation (70–73). Huang and colleagues irradiated four human cancer cell lines with photons, protons, and carbon ions. The results showed that 48 hours after irradiation, carbon ions at 2 Gy and 4 Gy significantly promoted the cell surface translocation of calreticulin, which was much more pronounced than with protons and photons (74). In normal cells, calreticulin is primarily found in the endoplasmic reticulum. When exposed on the cell surface, it can be recognized, taken up and processed by DCs, thus upregulating the immunogenic death of the tumor. This makes the irradiated tumor serve as an *in-situ* vaccine (75). Thus, carbon ion beams induce tumor cell death in various ways and release pro-inflammatory cytokines, chemokines, and express tumor antigens, referred to as Damage-Associated Molecular Patterns (DAMP). DAMPs can mediate the infiltration of immune cells in the tumor region, thereby activating the immune system (76).

Seventy years ago, the term “abscopal effect” was established by Mole and began to gain attention. This refers to the phenomenon where irradiating a tumor can lead to the regression of distant non-irradiated metastatic tumors. The biological mechanism behind this is not yet fully understood, but it is related to the immune system (77, 78). RT can trigger the release of new antigens (Tumor-Associated Antigens, TAA) from irradiated dying tumor cells, acting as *in-situ* vaccines. Once TAAs are phagocytosed by APCs, they are presented to CD8^+^ T cells. Activated CD8^+^ T cells can recognize and attack the primary tumor and distant metastatic tumors. Irradiated tumor cells can also release the aforementioned DAMPs and cytokines, further enhancing the activation of immune cells (79). However, the abscopal effect induced by RT is not common (80). Notably, some studies have confirmed that immunotherapy can enhance the radiotherapy-induced abscopal effect. In Grimaldi’s research, 21 patients with advanced melanoma underwent RT after ICIs (Ipilimumab) treatment. The results showed that 11 patients (52%) observed remission of distant tumors. The median OS for all 21 patients was 13 months, with the median OS being 22.4 months for those with the abscopal effect and only 8.3 months for those without (81). Komatsu reported a primary lung cancer patient treated with ICIs. After three weeks of treatment with Nivolumab, the tumor increased. Immediate RT was administered, showing a significant reduction in the irradiated tumor and also a decrease in the non-irradiated lung metastasis (82). The enhancement of the radiotherapy-induced abscopal effect by immunotherapy indicates the potential of this combined treatment approach. Further exploration is needed to understand the specific mechanisms of the radiotherapy-immunotherapy strategy, as well as determining the best dosages, intervention times, etc., to achieve optimal tumor therapeutic outcomes and effective treatment of cancer metastasis.

Carbon ions have been shown to be more effective than CRT in inducing the abscopal effect. In one case, a patient with recurrent thymic carcinoma showed a significant reduction in both the irradiated tumor and the non-irradiated distant tumor on a CT scan on day 1 after only receiving CIRT (60 Gy/12 fractions). Acute adverse reactions were limited to grade 1 radiodermatitis and mild erythema, with no adverse reactions greater than grade 2 observed, and no late adverse reactions found within 10 months (83). In another study, 3 out of 5 patients with assessable distant tumors observed the abscopal effect. Among them, two had multiple metastatic lesions, and all metastatic lesions achieved a tumor volume reduction, with an average volume reduction of 42% (84). Additionally, animal experiments have shown that CIRT is more effective than CRT in inhibiting the number of metastatic tumors in radiation-induced abscopal effect (85). In 4T1 tumor-bearing mice, both the irradiated and non-irradiated tumors’ growth was significantly delayed compared to the control group after receiving CIRT, indicating the direct and abscopal anti-tumor effects of carbon ions (86). However, the abscopal effect induced by either CRT or CIRT is not common (87). But when combined with immunotherapy like ICIs, significant control of both primary and distant tumors was observed, indicating the therapeutic potential of combined treatments in advanced tumors (85, 88).

In recent years, with research into the immunomodulatory effects of RT, a substantial body of evidence has demonstrated the effectiveness of combined RT and immunotherapy in cancer treatment. CIRT, based on its unique physical and biological advantages, has shown significant promise in combination therapy and holds potential for the treatment of certain radiation-resistant tumors and immunologically “cold” tumors.

In animal experiments involving the combination of carbon ion beams with ICIs, Zhou et al. irradiated subcutaneous melanomas in C57BL/6 mice with 5 Gy of X-rays and carbon ion beams. They administered anti-PD-1 treatment on days 1, 2 and 4 after irradiation. The results showed that in the combined treatment, CIRT promoted more exposure of calreticulin and the release of HMGB1 compared to XRT. It also induced a stronger IFN-1 response, demonstrating that CIRT effectively triggers immunogenic cell death. In the CIRT combined with anti-PD-1 group, there was an increase in the infiltration of CD4^+^ and CD8^+^ T cells into tumor tissues, leading to significant tumor control and an extended survival period for tumor-bearing mice (89). Guo et al. reported similar outcomes, showing that compared to X-ray, the combination of CIRT with anti-PD-L1 significantly increased the infiltration of CD4^+^, CD8^+^ T cells and NK cells into tumors, while delaying melanoma growth. Additionally, CIRT up-regulated the expression of PD-1 on the surface of immune cells, whereas XRT did not. Furthermore, CIRT induced the exposure of PD-L1 on tumor cells. High PD-1 expression in tumor-infiltrating immune cells is considered a characteristic of immunologically “hot” tumors (63, 90). Thus, these results demonstrate the synergistic effects of carbon ion beams combined with anti-PD-L1 (or anti-PD-1) in cancer therapy.

In a mouse osteosarcoma model, both CIRT and XRT, in combination with two ICIs (anti-PD-1 and anti-CTLA-4), inhibited the growth of distant tumors and lung metastasis. The combination of CIRT and ICIs showed the most significant effect, while monotherapy with radiation or ICIs did not achieve control of distant tumors (85).In another mouse breast cancer model, the combination of CIRT with ICIs achieved even more remarkable therapeutic effects. Analysis of tumors irradiated with carbon ion beams and treated with anti-CTLA-4 showed activation of NK cells, up-regulation of tumor-associated macrophages, TNF and IL-1 response genes, along with improved activity of naive T cells in distant non-irradiated tumors. Furthermore, cured mice exhibited long-lasting anti-tumor immunity (14). These results indicate that combination therapy effectively optimizes the TME, which may be one of the reasons for distant effects and specific tumor immune memory. In RT combined with DC-based therapy, after CIRT and XRT combined with intratumoral or intravenous DC administration in mice, the former significantly inhibited lung metastasis of mouse tumors when combined with DC, while the latter required higher doses to achieve lung metastasis suppression. Compared to XRT, CIRT significantly up-regulated the exposure level of extracellular calreticulin in tumor cells (91). Therefore, CIRT may promote the maturation of DC by increasing the immunogenicity of tumors, further stimulating anti-tumor immunity and reducing lung metastasis.

In terms of clinical trials, there are currently two ongoing clinical trials regarding carbon ion radiation combined with immunotherapy. One is a Phase II clinical trial combining CIRT and pamrelizumab for nasopharyngeal carcinoma patients after chemotherapy, with a plan to recruit 146 nasopharyngeal carcinoma patients (NCT04143984). The other is a multi-center Phase II clinical trial planned to include 27 patients (NCT05229614), using large fraction CIRT and pembrolizumab to treat non-small cell lung cancer, head and neck squamous cell carcinoma, melanoma, and urothelial carcinoma; results from both clinical trials have not yet been published.

## 5 Conclusions and perspectives

As research into radiotherapy and immunotherapy progresses, the synergistic effects of radiotherapy and immunotherapy become more apparent, especially for tumors that are not sensitive to conventional photon radiotherapy. Carbon ions can effectively kill tumors resistant to XRT, such as RCC, and exert an immune-activating effect. The synergistic effects in anti-tumor of this combined method offer the hope of eliciting a durable and potent immune response against tumors and having a new method of cancer treatment. Although preclinical and clinical research have shown the advantages and efficacy of CIRT. However, research combining carbon ions with immunotherapy are still rare and have not been reported in renal cell carcinoma. This may be due to the fact that heavy ion accelerators are not yet widely available globally and the high cost of treatment limits related research.

There are possible risks and problems associated with the combination therapy. Adverse reactions such as radiation pneumonitis and dermatitis also accompany carbon ion irradiation but are generally comparable or milder than CRT. Over-activation of the immune system may cause systemic toxicity and damage the body. With the optimization of accelerators, the cost of CIRT will be lower in the future, and more cancer patients can receive this advanced treatment. And new RT technologies, such as SBRT and Flash RT, are constantly emerging, which can control the radiation dose distribution in the tumor target more accurately, further improve the efficacy of carbon ion therapy and reduce adverse reactions. The adverse reactions of tumor immunotherapy are milder and usually reversible than those of conventional chemotherapy, and most patients can benefit from it. The potent immune-activating effect of CIRT is expected to reduce the dose of immunotherapeutic agents, thereby further minimizing the adverse reactions. In the future, CIRT will benefit more cancer patients, and its combination with other treatment methods, such as immunotherapy, is expected to achieve the ultimate goal of cancer treatment from “remission” to “cure”.

## Author contributions

ZZ: Conceptualization, Investigation, Visualization, Writing – original draft, Writing – review & editing. TY: Conceptualization, Writing – original draft. YL: Conceptualization, Funding acquisition, Writing – original draft. PQ: Writing – review & editing. ZS: Writing – review & editing. YW: Writing – review & editing. WC: Writing – review & editing. SU: Writing – review & editing. WW: Supervision, Writing – review & editing, Conceptualization, Funding acquisition, Project administration. ND: Funding acquisition, Project administration, Supervision, Writing – review & editing. JW: Supervision, Writing – review & editing.
